# Plan de negocio de intervenciones de enfermería: Programa “Cuidando a los Cuidadores”*

**DOI:** 10.15649/cuidarte.1994

**Published:** 2022-10-15

**Authors:** Yaira Pardo-Mora, Lorena Chaparro-Díaz, Sonia Carreño-Moreno

**Affiliations:** 1 . Doctora en Enfermería. Profesora Asistente, Facultad de Enfermería, Universidad Nacional de Colombia. Bogotá, Colombia. Email: yypardom@ unal.edu.co Universidad Nacional de Colombia Facultad de Enfermería Universidad Nacional de Colombia Bogotá Colombia yypardom@ unal.edu.co; 2 . Doctora en Enfermería. Profesora asociada, Facultad de Enfermería, Universidad Nacional de Colombia. Bogotá, Colombia. Email: olchaparrod@ unal.edu.co Universidad Nacional de Colombia Facultad de Enfermería Universidad Nacional de Colombia Bogotá Colombia olchaparrod@ unal.edu.co; 3 . Doctora en Enfermería. Profesora asociada, Facultad de Enfermería, Universidad Nacional de Colombia. Bogotá, Colombia. Email: spcarrenom@ unal.edu.co Universidad Nacional de Colombia Facultad de Enfermería Universidad Nacional de Colombia Bogotá Colombia spcarrenom@ unal.edu.co

**Keywords:** Costos y Análisis de Costo, Cuidadores, Enfermedad Crónica, Cuidado de Enfermería, Costs and Cost Analysis, Caregivers, Chronic Disease, Nursing Care, Custos e Análise de Custos, Cuidadores, Doengas Crónicas, Cuidados de Enfermagem

## Abstract

**Introducción:**

En Colombia el 65,51% de las atenciones prestadas fueron por enfermedades crónicas no transmisibles. Estas generan algún grado de discapacidad y dependencia, requiriendo un cuidador.

**Objetivo:**

Establecer una propuesta de plan de negocio para el programa "Cuidando a los cuidadores”, orientada al análisis de costos, económico y financiero.

**Materiales y Métodos:**

Estudio de evaluación económica de un programa de enfermería, con análisis de costos el cual se llevó a cabo en 4 pasos: 1) Caracterizar la estructura de costos del programa, 2) Elaborar las proyecciones, 3) Establecer el valor comercial de los niveles del programa, y 4) Evaluar mediante indicadores financieros la información obtenida en los pasos anteriores, para determinar la viabilidad económica del programa.

**Resultados:**

Se estima un costo anual de $6,9115 pronosticando a 5 años un costo anual de $8,732 en el nivel básico y de $7,131 en el nivel de seguimiento del programa. Los recursos para la implementación del programa son de $6,101, presentando un incremento patrimonial de $12,177 entre 2019 y 2023. Finalmente, la tasa interna de retorno- TIR es de 326,19%, lo que indica que por cada $1 invertido, retornarán $326, concluyendo que el programa financieramente es rentable.

**Discusión:**

Se realizó el análisis únicamente del programa "Cuidando a Cuidadores®”, debido a que no existen en Colombia programas con características similares.

**Conclusión:**

"Cuidando a los cuidadores”, es un programa con un potencial de crecimiento en Colombia, debido al beneficio social que ofrece, y a la escaza competencia que presenta. La evaluación financiera demostró que es rentable.

## Abstract (en)

**Introduction:**

In Colombia, 65.51% of the care provided was for chronic non-communicable diseases. These generate some degree of disability and dependency, requiring a caregiver.

**Objective:**

Establish a business plan proposal for the “Caring for caregivers” program, oriented to cost, economic and financial analysis.

**Materials and Methods:**

Economic evaluation study with a cost analysis of a nursing program in which four steps were followed for data analysis: 1) Describe the cost structure of the program, 2) make projections, 3) determine the commercial value of levels offered by the program, and 4) evaluate the information obtained in the previous steps using financial indicators to determine if the proposal makes the program economically viable.

**Results:**

An average annual cost of $ 6.9115 is estimated, forecasting a 5-year average annual cost of $ 8732 at the basic level and $ 7131 at the program monitoring level. The resources for the implementation of the program are $ 6.101, presenting an equity increase of $ 12.177 between 2019 and 2023. Finally, the internal rate of return - IRR is 326.19%, this means that for every $ 1 invested in the program, they will return $ 326 concluding that the program from this financial point of view is profitable.

**Discussion:**

Only the "Caring for Caregivers" program was analyzed, because there are no programs with similar characteristics in Colombia.

**Conclusion:**

"Caring for caregivers®" is a program with growth potential in Colombia, due to the social benefit it offers, and the scarce competition it presents. The financial assessment showed that it is cost-effective.

## Resumo

**Introdujo:**

Na Colombia, 65,51% dos atendimentos foram por doencas crónicas nao transmissíveis. Estas geram algum grau de deficiencia e dependencia, requerendo um cuidador.

**Objetivo:**

Estabelecer uma proposta de plano de negócio para o programa "Cuidando dos cuidadores', orientada a análise de custos, económico e financeiro.

**Materiais e Métodos:**

Estudo de avaliacáo económica com análise de custos de um curso de enfermagem em que foram realizadas quatro etapas para análise de dados: 1) Caracterizar a estrutura de custos do curso, 2) Elaborar projecóes, 3) Estabelecer o valor comercial dos níveis oferecidos pelo programa, e 4) Avaliar por meio de indicadores financeiros as informales obtidas nas etapas anteriores, para determinar se a proposta torna o programa economicamente viável.

**Resultados:**

Estima-se um custo médio anual de $ 6,9115, prevendo um custo médio anual de 5 anos de $ 8.732 no nível básico e $ 7.131 no nível de acompanhamento do programa. Os recursos necessários para a implementacáo do programa sáo de $ 6.101, apresentando um aumento de ativos de $ 12.177 entre 2019 e 2023. Por fim, a taxa interna de retorno - TIR é de 326,19%, isso significa que para cada $ 1 investido no programa, eles seráo retornar $326, concluindo que o programa é lucrativo deste ponto de vista financeiro.

**Discussao:**

Foi realizada a análise unicamente do programa "Cuidando a Cuidadores®", devido a que náo existem na Colombia programas com características similares

**Conclusao:**

"Cuidar de cuidadores®" é um programa com potencial de crescimento na Colómbia, pelo benefício social que oferece e pela falta de concorrencia que apresenta. As despesas operacionais versus as receitas que gera mostraram que é financeiramente rentável.

## Introducción

De acuerdo con la Organización Mundial de la Salud OMS (1) para el año 2016 se calculó que, de los 56,4 millones de defunciones registradas, más del 54% estaban relacionadas con enfermedades crónicas no trasmisibles (ECNT). En Colombia, a partir de los Registros Individuales de Prestación de Servicios (RIPS), entre 2009 y 2017 el 65,51% de las atenciones prestadas fueron por enfermedades crónicas no transmisibles^1^^,^^2^. Las ECNT generan algún grado de discapacidad y dependencia^3^, por lo que la mayor parte de estas personas requiere de un cuidador.

La necesidad de un acompañamiento constante para el manejo de la enfermedad e incluso para su autocuidado, hace evidente el requerimiento de una persona que asuma el rol de cuidador familiar^4^. El cuidador familiar generalmente tiene un vínculo de parentesco o cercanía con la persona con ECNT, y al decidir asumir la responsabilidad del cuidado en casa, se transforma toda su vida y su cotidianidad, viéndose enfrentado a diversas pérdidas, como consecuencia del tránsito de roles, situación que frecuentemente se asocia con la percepción de sobrecarga e inestabilidad emocional^5^^-^^7^.

Ante esta realidad de los cuidadores en Colombia, nace el programa “Cuidando a los cuidadores”, del grupo de investigación de Cuidado de Enfermería al Paciente Crónico, de la Facultad de Enfermería de la Universidad Nacional de Colombia que ofrece este programa a los cuidadores familiares de personas con enfermedad crónica que contactan el servicio de manera directa o por remisión de profesionales, asociaciones u otros servicios. Este programa fue oficializado como proyecto de extensión solidaria desde el año 2005; y se han vinculado aproximadamente a 2.000 usuarios, quienes han logrado un reconocimiento social y familiar frente a su labor, se han fortalecido en la habilidad de cuidado, recibido capacitación en actividades instrumentales para el cuidado e incursionado en el uso de la tecnología, con miras a la conformación de una red de apoyo social^8^^,^^9^, logrando en el 2019 el registro de marca ante la Superintendencia de Industria y Comercio de Colombia (Resolución 2684 de 2019. Expediente No. SD2018/0062855).

En el marco de la realización del registro en industria y comercio, se realizó un análisis de costos del programa con el se pretendió conocer su valor económico a fin de evidenciar su comportamiento financiero y determinar su viabilidad en términos de rentabilidad. Por lo anterior, se hace una evaluación económica que tiene como objetivo establecer una propuesta de plan de negocio para el programa “Cuidando a los cuidadores”, desarrollando un análisis del contexto, un análisis de costos, un análisis económico y un análisis financiero-basados en la información de los años 2016 al 2018 y realizando una proyección a 5 años desde el año 2019.

En Colombia existen pocos estudios de costos de programas en salud^10^, por lo que este análisis representa una forma de definir la viabilidad de programas similares en términos de operación y de sostenibilidad financiera de los mismos, en países como Colombia donde los proyectos tienen escasos recursos institucionales y acceso restringido a fuentes de financiación externa.

## Materiales y Métodos

El tipo de estudio realizado fue una evaluación económica con análisis de costos^11^ cuyo objetivo es realizar un análisis descriptivo de los costos y rentabilidad actual y proyectada del programa “Cuidando a Cuidadores” de la Facultad de Enfermería de la Universidad Nacional de Colombia. Para esto se establecieron las siguientes variables metodológicas:

*Variable 1.* Rentabilidad plan de negocio para el programa “Cuidando a los cuidadores”, esta permite establecer la proyección y rentabilidad con beneficios para la sociedad.
*Variable 2.* Caracterización del programa (proyección indicadores financieros): Para el análisis de rentabilidad se verificaron valores de inversión, costos, la trazabilidad de indicadores financieros que determinaron los beneficios y aspectos que pueden ser contrarios a los objetivos propuestos.
*Variable 3.* Alto potencial de crecimiento en Colombia, por ser un beneficio social.

Para el análisis se tuvo en cuenta la normatividad institucional bajo el cual funciona el programa en la Universidad entre los cuales se tuvieron en cuenta:

Detalle del costo de variables como el alquiler de salones, gastos papelería y suministros, impuestos, tasas y contribuciones y transferencias.
Normas de ejecución para los proyectos de extensión en la Universidad Nacional de Colombia.
Lista de integrantes del programa (docentes, estudiantes y expertos), teniendo en cuenta su vínculo con la universidad. Si eran profesores vinculados, se relacionaba el valor de la hora de acuerdo, a su ingreso salarial, en el caso de estudiantes y expertos se estimó un valor promedio en proyectos desarrollados por el grupo de investigación.
Numero de talleres ofertados y desarrollados en el periodo, detallando la dedicación en horas.
Número y horas de uso de salones para su desarrollo de las sesiones
Material entregado a los cuidadores para el desarrollo de las sesiones.

Esta evaluación se realizó en cuatro fases, que se describen a continuación:

1. *Caracterización de la estructura de costos del programa:* en esta se identifican materiales directos, mano de obra directa y costos indirectos de fabricación, los cuales determinan el costo de producción de un bien o servicios^12^
2. *Elaborar proyecciones:* esta debe estar enfocada en los planes a futuro, involucrando las condiciones económicas como el endeudamiento, la situación de liquidez, la naturaleza de las transacciones es financieras y la movilización o rotación de fondos invertidos^13^. Aquí también se debe conocer las operaciones económicas realizadas desde el inicio del programa hasta el desarrollo de la propuesta
3. *Establecer el valor comercial de los niveles que ofrece el programa:* se realiza con un análisis profundo de los costos y expectativas de la rentabilidad que se tiene. Para esto es necesario investigar los métodos de fijación de precios más adecuado para los servicios que el programa presta.
4. Evaluar mediante indicadores financieros la información obtenida para determinar si la propuesta hace que el programa sea económicamente viable. Aquí se debe elegir el valor comercial de cada nivel que presta el programa y ver cómo impacta el desarrollo del mismo, así como la elaboración del presupuesto.

Este análisis hizo parte de un proyecto de extensión solidaria financiado por la Universidad Nacional de Colombia en el que se pretendía generar escalabilidad de modelos de Extensión Solidaria que pretenden proyectar el impacto de programas existentes.

## Resultados

A continuación, se describe en detalle el análisis de costos actuales y proyectados, el análisis económico y el análisis financiero, con los cuales se determinó la rentabilidad del programa versus los costos que genera su desarrollo. La base de datos fue almacenada en Mendeley Data^14^.

### 1. Análisis de costos

En este se tuvieron en cuenta los costos directos, costos indirectos y gastos administrativos. En el estudio se identificó que el valor de las horas docentes tiene el mayor peso en los costos directos y los refrigerios que se dan a los cuidadores y el uso de salones del edificio donde funciona el programa en los costos indirectos. En cuanto a los gastos administrativos, este representa el costo mayor porque implica el soporte administrativo que requiere el programa por estar vinculado a una Institución de Educación Superior. La estructura de costos se detalla en la Tabla 1.

**Tabla 1:** Estructura de costos actual del programa “Cuidando a los cuidadores”

Costos Directos Operativos (A)	10,8%
Docentes Nivel Básico	6,5%
Docentes Nivel de seguimiento	1,1%
Estudiantes de posgrado	0,0%
Equipo de profesionales	0,9%
Contratistas	1,5%
Cartillas	0,8%
Costos Indirectos de Operación (B)	5,5%
Alquiler Salones y Auditorios Nivel Básico	0,8%
Alquiler Salones y Auditorios Nivel de seguimiento	1,3%
Alquiler Clausura	0,6%
Refrigerios	2,1%
Constancias	0,4%
Insumos oficina	0,4%
Gastos Administrativos (C)	83,7%
4 x 1000	0,1%
Personal administrativo	46,7%
Consultas legales	12,8%
Diseños gráficos	9,4%
Imprevistos	0,8%
Depreciación	0,2%
Licencia	0,1%
Página web	0,4%
Registro de marca	0,1%
Gastos de viaje	0,5%
Suministros papelería y artículos de oficina	0,2%
Aseo, cafetería y vigilancia	5,3%
Gestión de bienes	2,6%
Alquiler de oficina	3,4%
Impuestos, tasas y contribuciones	0,0%
Materiales, suministros e insumos	0,0%
Servicios públicos	1,0%
Otros costos	0,1%
Total Costos	100,0%

Al recopilar los informes de ejecución de los años 2016 a 2018, se estima un costo anual en promedio de $262.640.356,37 pesos colombianos (69.116 dólares aproximadamente, con un valor del dólar de $3.800 pesos colombianos que adelante se utilizará como TRM para los valores incluidos en las tablas). La Tabla 2 detalla los costos totales del programa en este rango de tiempo.

**Tabla 2:** Costos totales del programa “Cuidando a los cuidadores”

	2016	2017	2018
Costos Directos Operacionales	$ 5.576,94	$ 9.229,81	$ 7.665,06
Costos Indirectos de Operación	$ 3.147,51	$ 3.811,94	$ 4.362,90
Gastos Administrativos	$ 51.715,67	$ 63.569,08	$ 58.266,46
Costos totales	$ 60.440,11	$ 76.610,83	$ 70.294,42

### 2. Análisis económico

Frente a la inversión inicial del programa, esta contempló los bienes depreciables y físicos que son utilizados dentro de las actividades principales del programa. La depreciación se realizó bajo en modelo de línea recta y el tiempo de vida útil (Artículo 82 de la Ley 1819 de 2016 de Colombia) determinando que el total de inversión en activos depreciables es de $10.299.000 pesos colombianos (2656,77 USD).

En los costos de operación pronosticados, se tuvo en cuenta los materiales (cartillas del programa), la mano de obra directa representada en el valor/hora de docentes, los equipos de profesionales, estudiantes de pregrado y posgrado. Se estimó en 5 años un promedio de costos anuales por $33.179.186 (8559,02USD) en el nivel básico y de $27.100.211 (6990,86 USD) en el nivel de seguimiento del programa. De manera discriminada, para el año 2019 se pronostica un costo operacional total de $55.645.722 (14354,56 USD), para el 2020 de $57.871.717 (14928,78 USD), para el 2021 de $60.168.759 (15521,34 USD), para el 2022 de $62.594.410 (16147,07 USD) y para el 2023 de $65.098.374 (16793 USD), con una diferencia proyectada de $9.452.652 (2438,44 USD) de incremento de costos a 5 años.

Respecto a los gastos administrativos del programa se tuvo en cuenta que el mayor gasto promedio es de $517.097.395 (133.392 USD). Entre los costos que más ocupan esta proyección están las consultas legales asociadas al registro de marca del programa que se inició en 2017 y fue otorgado por la Superintendencia de industria y comercio en 2019 por 10 años (Registro de marca No. 615885 Resolución 2684 de 2019. Expediente No. SD2018/0062855) y el personal involucrado como docente coordinador, el apoyo administrativo y el recurso de diseño gráfico que se requiere para la actualización de las cartillas y piezas divulgativas del programa. Esta información se precisa en la Tabla 3.

**Tabela 3:** Informales sobre a internado dos pacientes submetidos a procedimento neurológico, Porto Velho/RO, 2018-2019 (n=36)

Costo o Gasto	2019	2020	2021	2022	2023
Coordinador(a) General	$ 47134,39	$ 48736,96	$ 50296,54	$ 51931,18	$ 53535,85
Profesional Administrativo	$ 49.882,46	$ 51.578,47	$ 53.228,98	$ 54.958,92	$ 56.657,15
Apoyo Administrativo	$ 4.134,95	$ 4.275,54	$ 4.412,35	$ 4.555,75	$ 4.696,53
Alquiler Oficina	$ 2.363,34	$ 2.443,69	$ 2.521,89	$ 2.603,85	$ 2.684,31
4 X 1000	$ 44,17	$ 45,67	$47,13	$48,67	$50,17
Consultas Legales	$8.664,66	$8.959,26	$9.245,96	$9.546,45	$9.841,44
Diseñador Gráfico	$6.370,90	$6.587,51	$6.798,31	$7.019,26	$7.236,16
Imprevistos	$552,12	$570,90	$589,17	$608,31	$627,11
Licencia	$257,96	$266,73	$275,27	$284,22	$293,00
Página Web	$806,13	$833,54	$860,21	$888,17	$915,61
Registro De Marca	$201,53	$208,38	$215,05	$222,04	$228,90
Gastos De Viaje	$343,95	$355,65	$367,03	$378,95	$390,66
Suministros Papelería Y Artículos De Oficina	$119,37	$123,43	$127,38	$131,52	$135,59
Aseo, Cafetería Y Vigilancia	$3.562,04	$3.683,15	$3.801,01	$3.924,54	$4.045,81
Gestión De Bienes	$1.747,66	$1.807,08	$1.864,91	$1.925,51	$1.985,01
Impuestos, Tasas y Contribucio nes	$19,30	$19,96	$20,60	$21,27	$21,92
Servicios Públicos	$679,70	$702,81	$725,30	$748,88	$772,02
Otros Costos	$87,72	$90,70	$93,60	$96,64	$99,63
Total Gastos Administrativos	$124.930,16	$129.177,79	$133.311,48	$137.644,10	$141.897,30

Para el cálculo de los costos de financiación pronosticados se tuvo en cuenta la inversión líquida que se debe aportar para que el programa funcione, en este caso se estimó el desarrollo de los niveles básico y de seguimiento de manera anual. A partir de esto, los recursos necesarios para la puesta en marcha del programa son de $23.185.717 (5.981 USD), diferenciados en capital para mano de obra en $9.881.598 (2549 USD), para materiales en $739.247 (190,70 USD) y para costos indirectos en $12.564.873 (3241 USD). Además, se tuvo en cuenta el costo de capital con una tasa de interés efectiva mensual de 1,2%, como promedio de las tasas de interés encontradas en algunos bancos del sector financiero colombiano.

Para los costos pronosticados en total y por nivel, se estimaron de forma anual por $960.327.218 (250.869 USD) para el periodo 2019-2023 y en cuanto al costo por nivel y por persona se estimó en $2.88.202 (754 USD) para el nivel básico y $1.444.101 (377 USD) para el nivel de seguimiento. Esta información se especifica en la Tabla 4.

**Tabla 4:** Costo unitario por nivel pronosticado de “Cuidando a los cuidadores”

Periodo	2019	2020	2021	2022	2023
Costo por persona nivel básico y seguimiento + margen transf. 39%	$1.049	$1.083	$1.117	$1.152	$1.187
Costo por persona nivel básico + margen transf. 39%	$699	$722	$745	$768	$791
*Costo por persona nivel de seguimiento + margen* transf. 39%	$350	$361	$372	$384	$396

### 3. Análisis Financiero

La proyección de los ingresos del programa se realizó bajo el costo por persona más el margen de las trasferencias de la Tabla 4, teniendo en cuenta las proyecciones con respecto a la inflación, para los 5 años desde el año 2019. Estos valores pueden ser indicadores para la venta de servicios basados en la rentabilidad anual proyectada como se muestra en la Tabla 5.

**Tabla 5:** Ingresos proyectados para el programa “Cuidando a los cuidadores”

	2019	2020	2021	2022	2023
Nivel Básico*	$143.347	$148.034	$152.630	$157.450	$162.216
Nivel de seguimiento**	$89.155	$92.070	$94.928	$97.926	$100.890
Total	$232.501	$240.104	$247.558	$255.377	$263.106

* Primer semestre 118 usuarios, segundo semestre 87 usuarios (promedios encontrados en informes 2016-2018) ** 255 usuarios al año (promedios encontrados en informes 2016-2018)

Para el estado de situación financiera proyectado se analizó el comportamiento de los activos, los pasivos, y el patrimonio del programa según las proyecciones de los anteriores ítems. En activos totales, se identificó un promedio proyectado de 383.301.875 (98877 USD), con un valor para el 2019 de $370.053.767 (95.460 USD) y para el 2023 de $397.775.582 (102.611 USD), mostrando una diferencia de 27.721.815 (7151 USD). Para los pasivos, se espera una disminución gradual de los mismos en los siguientes 5 años, iniciando con $18.548.574 (4.784 USD) en 2019 y quedando en ceros para 2023. Finalmente, se espera un incremento patrimonial de 46.270.389 (11.936 USD) para un valor de $397.775.582 (102.611 USD) para el 2023.

En el estado de resultados se puede observar la proyección de los ingresos, costos y gastos del programa, teniendo en cuenta que el excedente final es un porcentaje fijo (34%) que corresponden a las transferencias de universidad y facultad bajo la normatividad vigente, quedando finalmente un porcentaje de 39%, aspecto que se detalla en la Tabla 6.

**Tabla 6:** Estado de resultados proyectado de “Cuidando a los cuidadores”

Estado De Resultados Proyectado	2019	2020	2021	2022	2023
Ingresos	$232.501	$240.104	$247.558	$255.377	$263.106
Costos De Operación	$14.355	$14.929	$15.526	$16.147	$16.793
Depreciación	$424	$424	$424	$424	$424
Excedente Bruto	$217.722	$224.751	$231.607	$238.805	$245.889
Gastos Administrativos	$124.930	$129.178	$133.311	$137.644	$141.897
Excedente Operativo	$92.792	$95.573	$98.296	$101.161	$103.992
Intereses	-$920	-$736	-$552	-$368	-$184
Servicio de la deuda	-$1.196	-$1.196	-$1.196	-$1.196	-$1.196
Excedente Neto Final (39%)	$90.675	$93.640	$96.548	$99.597	$102.612

El *Valor Presente Neto (VPN)*, como uno de los indicadores más utilizados para evaluar la viabilidad de proyectos que consiste en calcular los valores presentes netos con una tasa de oportunidad y que representa la tasa que se espera obtener del programa en el excedente total, correspondió al 39%.

Por último, la *tasa interna de retorno - TIR* para el programa fue de 326,19%, esto significa que por cada $1 invertido en el programa, retornarán $326. La tasa de 326,19% es bastante superior, por lo tanto, se concluye que el programa desde este punto de vista financiero es rentable.

## Discusión

Los estudios de evaluación económica apoyan la toma de decisiones en salud, representando un valioso mecanismo que mejora la eficiencia de los procesos de distribución presupuestaria entre los distintos niveles de atención en salud^9^^,^^15^. Generalmente, se comparan dos o más intervenciones para determinar cuál de ellas tiene los mejores efectos a menor costo, pero en este caso, se realizó el análisis únicamente del programa “Cuidando a Cuidadores®”, debido a que no existen en Colombia programas con características similares (trayectoria, contenidos, didácticas y enfoque teórico - disciplinar de enfermería), con los cuales hacer la comparación de costo-efectividad.

En contextos internacionales, se han desarrollado evaluaciones económicas similares, enfocadas a presentar un plan de negocios respecto a las intervenciones de cuidado diseñadas por profesionales de enfermería. Al respecto, diversos autores^16^^-^^17^ enfocaron las intervenciones en el desarrollo de centros día u hogares de cuidado para adultos mayores de diferentes culturas, encontrando que a pesar de que varios de ellos eran programas sin ánimo de lucro, sus ingresos estaban vinculados a donaciones, aportes del estado y subsidios de empresas privadas, y con ellos asumían los gastos anuales vinculados a la atención de estas personas. Todas estas intervenciones demostraron que estos enfoques de cuidado culturalmente congruentes son rentables y mejoran no solo la calidad de vida de estas personas, sino también de sus cuidadores.

Comparando estas intervenciones con el programa “Cuidando a Cuidadores”, se evidencia la capacidad de innovación que tienen los profesionales de enfermería para plantear y desarrollar actividades que mejoren la salud y la calidad de vida de las personas, resaltando la capacidad que demuestran para aplicar el conocimiento teórico a la práctica por medio de intervenciones, que representan tecnologías blandas a bajo costo y con alto impacto social. Esto ratifica que es indispensable la presencia de enfermería dentro de los equipos de salud, dado que sus acciones, especialmente en el contexto de la práctica, garantiza la disminución de costos en los servicios de salud^18^.

Para Vargas^19^ el análisis de beneficios y costos permite realizar una evaluación económica de los programas en general en salud, por tanto, identificar cuánto cuestan y si son rentables o no puede mostrar la utilidad y aplicaciones de metodologías como la del presente estudio para demostrar a futuros fuentes de financiación el impacto de invertir en programas como el de “Cuidando a Cuidadores”. Es así como por ejemplo en un estudio realizado para una unidad de gastroenterología, en el que enfermería era el orientador y principal asesor de pacientes y cuidadores que accedieron al servicio de endoscopia se identificó una mejora en los ingresos de la unidad, dado que disminuyó en un 10% la cancelación de procedimiento y la inasistencia al mismo, lo que permite inferir que los ingresos proporcionan una rentabilidad a largo plazo.

Los profesionales de enfermería requieren comprender la importancia de los análisis económicos, los cuales posibilitan la evidencia para la ejecución de intervenciones que se planean desde la teoría, la práctica y la evidencia empírica^20^. Como se precisa en este plan de negocios, las intervenciones de enfermería son rentables y tienen un alto valor social que contribuye al bienestar de los sujetos de cuidado, dado que muchas de ellas son actividades sin ánimo de lucro, pero su aporte a la salud de las poblaciones resalta el valor que tienen, tal como el programa “Cuidando a Cuidadores”.

Las limitaciones de este estudio se relacionan principalmente con la disponibilidad de estudios económicos en enfermería a nivel de Latinoamérica con los cuales se pueda contrastar el plan de negocios presentado. Además de esto, no se encontraron intervenciones para cuidadores con principios similares con el fin de haber desarrollado una comparación en costo efectividad de estas.

## Conclusión

“Cuidando a Cuidadores”, es un programa con un potencial de crecimiento en Colombia, esto debido al beneficio social que ofrece, y a la escaza competencia que aún conserva. El presente estudio aborda una primera mirada para comprender su funcionamiento, y poder llegar a un valor comercial, tanto del nivel básico como del nivel de seguimiento; no obstante, el escaso material disponible fue una limitante en el desarrollo del trabajo.

Sin embargo, en una primera aproximación, el programa presenta un valor agregado a los servicios que las entidades prestadoras de salud ofrecen no sólo a los pacientes, sino también a los cuidadores de personas con enfermedades crónicas no transmisibles, dentro de las cuales, están las principales causas de muerte en Colombia. Esto podría ser un avance en términos de responsabilidad social y gobierno corporativo.

El programa “Cuidando a Cuidadores” ha tenido una trayectoria importante en diferentes escenarios clínicos e institucionales, su aporte a la docencia e investigación permite identificar las necesidades de los cuidadores en diferentes contextos sociales, permitiendo que sean más las personas las beneficiadas con este programa. El impacto del programa ha servido de modelo para ser replicado y adaptado a diferentes contextos ambulatorios y hospitalarios, pues contribuye a disminuir la carga, mejorar la habilidad de cuidado y permitir que la experiencia del rol de cuidador sea satisfactoria ^17^^-^^19^.

Se vislumbra con la investigación, que existe un alto crecimiento y desarrollo en la economía en el sector explorado, que alcanza a obtener índices altos y rentables para los creadores del proyecto.
